# Molecular and phylogenetic analysis of HIV-1 variants circulating among injecting drug users in Mashhad-Iran

**DOI:** 10.1186/1750-9378-1-4

**Published:** 2006-09-19

**Authors:** HR Naderi, M Tagliamonte, ML Tornesello, M Ciccozzi, G Rezza, R Farid, FM Buonaguro, L Buonaguro

**Affiliations:** 1 Inst. of Infectious Diseases, Univ. of Mashhad –, Iran; 2 Viral Oncogenesis and Immunotherapy & AIDS Refer. Center, Ist. Naz. Tumori "Fond. G. Pascale", Naples –, Italy; 3 Epidemiology Unit, Dept. Infectious Diseases, ISS, Rome –, Italy; 4 Allergy and Immunology Dept, Univ. of Mashhad –, Iran

## Abstract

Genetic and phylogenetic information on the HIV-1 epidemic in Middle-East Countries, and in particular in Iran, are extremely limited. By March 2004, the Iranian Ministry of Health officially reported a cumulative number of 6'532 HIV positive individuals and 214 AIDS cases in the Iranian HIV-1 epidemic. The intra-venous drug users (IDUs) represent the group at highest risk for HIV-1 infection in Iran, accounting for almost 63% of all HIV-infected population. In this regards, a molecular phylogenetic study has been performed on a sentinel cohort of HIV-1 seropositive IDUs enrolled at the end of 2005 at the University of Mashhad, the largest city North East of Tehran. The study has been performed on both *gag *and *env *subgenomic regions amplified by Polymerase Chain Reaction (PCR) from peripheral blood mononuclear cells (PBMCs) and characterized by direct DNA sequence analysis. The results reported here show that the HIV-1 subtype A is circulating in this IDUs sentinel cohort. Moreover, the single phylogenetic cluster as well as the *intra*-group low nucleotide divergence is indicative of a recent outbreak. Unexpectedly, the Iranian samples appear to be phylogenetically derived from African Sub-Saharan subtype A viruses, raising stirring speculations on HIV-1 introduction into the IDUs epidemic in Mashhad. This sentinel study could represent the starting point for a wider molecular survey of the HIV-1 epidemics in Iran to evaluate in detail the distribution of genetic subtypes and possible natural drug-resistant variants, which are extremely helpful information to design diagnostic and therapeutic strategies.

## Background

The first case of HIV/AIDS in Iran was reported in 1987 in a 6-year-old child with hemophilia and since then, the number of HIV infections in Iran has increased exponentially within the hemophiliacs group [1]. After the introduction in 1989 of the mandatory blood (and its derivatives) screening for HIV-1 positivity, the HIV-1 spread among hemophiliacs has dramatically dropped and the intra-venous drug users (IDUs) have progressively become the group at highest risk for HIV-1 infection (62.8% of all reported infections) [1]. In particular, the needle sharing among inmate IDUs represents a specific high risk behavior for HIV-1 transmission in Iran [2,3]. Furthermore, in the last years the HIV infection is increasingly spread in the Iranian population through sexual route (7.3% of all reported cases), although a considerable number of infections (26.1%) are reported to be transmitted through unknown routes [4]. By March 2004, the Iranian Ministry of Health officially reported a cumulative number of 6'532 HIV positive individuals and 214 AIDS cases [5,6]. However, the latest report from UNAIDS estimates a number of HIV/AIDS cases in Iran which could be as much as four times higher than those officially registered [7].

Limited data are available on HIV-1 subtype distribution in the Middle East region where the B and C subtypes are prevalent, with the exception of Lebanon where the A subtype is predominant [8]. Molecular epidemiology studies are extremely important to know the HIV-1 subtype distribution in a specific population/region which may significantly influence the diagnostic and therapeutic strategies. In fact, a correlation between HIV-1 genetic subtypes and natural resistance to antiretroviral drugs, as well as efficiency of diagnostic serological and molecular tests, has been observed [9-12]. Moreover, the degree of cross-protection induced by vaccines, based on subtypes not predominant in the target population, is still a debated matter [13].

In this regards, a molecular phylogenetic study has been performed on a sentinel cohort of Iranian HIV-1 seropositive IDUs enrolled at the end of 2005 at the University of Mashhad, the largest city northeast of Tehran. Blood samples were obtained from 12 HIV-1 positive patients attending the Department of Infectious Diseases, Imam Reza General Hospital where the HIV-1 infection was diagnosed by immunological methods (ELISA, Western blot) and the CD4+ T-cell population counted. At the enrollment, all of them declared to live in Mashhad and to be IDUs, except one patient who reported a homosexual behavior. Some of them have spent few years in prison, representing an IDU specific high risk group within the Iranian HIV-1 epidemic [2,3]. The full designation of samples, according to WHO-proposed nomenclature, is MSH05.00XE or MSH05.00XG, where 05 stands for the year of study and E (or G) stands for *env *(or *gag*). For the sake of simplicity, however, in this paper the samples have been indicated only with the isolate's number (e.g., 001) (Table 1).

**Table 1:** Epidemiological and Clinical Characteristics of Mashhad samples.

**Sample**	**Risk exposure**	**Status**	**CD4**^+ ^(cells/μl)	**ARV**	**PCR *gag***	**PCR *env***
**MSH01**	IDU	Alive	428	Yes	Pos	Neg
**MSH02**	IDU	Alive	444	No	Pos	Pos
**MSH03**	IDU	Alive	111	No	Pos	Pos
**MSH04**	IDU	Alive	191	No	Pos	Pos
**MSH05**	Homo	Dead	n.a.	No	Neg	Pos
**MSH06**	IDU	Alive	400	No	Pos	Pos
**MSH07**	IDU	Alive	450	Yes	Pos	Pos
**MSH08**	IDU	Alive	408	No	Pos	Pos
**MSH09**	IDU	Alive	446	No	Pos	Neg
**MSH10**	IDU	Dead	n.a.	No	Pos	Pos
**MSH11**	IDU	Alive	133	Yes	Pos	Neg
**MSH12**	IDU	Alive	129	No	Pos	Pos

n.a, not available;

homo, homosexual;

IDU, injecting drug user.

DNA was extracted at the Imam Reza General Hospital from 5 × 10^6 ^peripheral blood mononuclear cells (PBMCs) by the QIAamp DNA blood kit (Qiagen, Alameda, CA – USA), according to the manufacturer's instructions. The quality of target DNA was verified by PCR amplification of p53 housekeeping cellular gene as routinely performed at the INT in Naples, prior to viral genes amplification [14,15].

The hypervariable C2-V5 region of the HIV-1 *env *gene (667 bp) and the fragment spanning the p24 and p7 region of the *gag *gene (460 bp) have been amplified from approximately 1 μg of purified DNA (corresponding to 1.5 × 10^5 ^cells) by nested PCR, as previously described [14,15]. The DNA nucleotide sequence analysis has been performed on uncloned PCR products to identify the prevalent viral quasispecie. Nucleotide sequences (appr. 300 nucleotides) were aligned using CLUSTAL W [16], with minor manual adjustments, and pairwise compared to HIV-1 reference standards of different subtypes available through the Los Alamos Database [17]. Sites with gaps in any of the sequences, as well as areas of uncertain alignment, were excluded from all sequence comparisons. Phylogenetic trees were constructed with the neighbor joining method [18] and the Tree View software application was used to draw dendrograms. Genetic distances were calculated with Kimura's two-parameter method [19].

All Mashhad samples were positive for the highly conserved p24 *gag *subgenomic region, with the exception of the 005 sample; on the contrary, only 7 out of 12 samples (58.3%) were positive for the C2-V5 *env *subgenomic region. An alternative primer pair (ED5-ED12) in the first amplification round and less stringent annealing conditions (45°C vs 55°C) yielded the amplification of the *env *subgenomic region from 2 additional samples, from the 5 previously negative ones (Table 1). The negative amplification results in *gag *(MSH05) or *env *(MSH01, 09, 11) subgenomic regions could be explained by a significant number of nucleotide substitutions or deletions in the primers' target sequence, resulting in an inefficient primer-to-target annealing.

**Figure 1:**
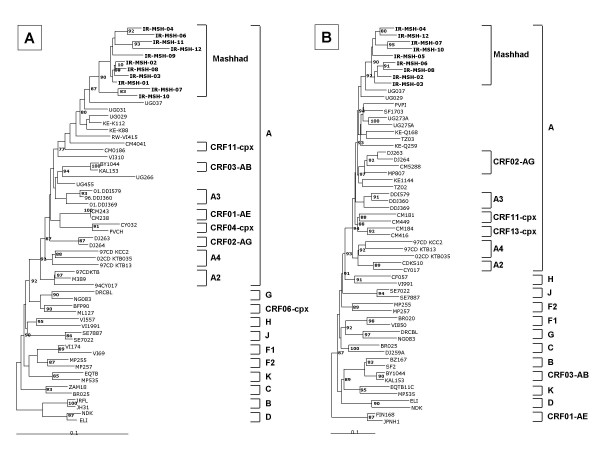
**Phylogenetic trees based on HIV-1 p24 gag (A) and C2-V3 env (B) regions**. Nucleotide sequences of Mashhad isolates are compared with HIV-1 reference strains of subtypes A – J of Group M. Trees have been constructed by the neighbor-joining method on 300 unambiguously aligned positions; the reliability has been estimated from 1'000 bootstrap replicates and values above 65% are indicated. The Mashhad sequences are indicated in bold characters.

The HIV-1 samples identified in Mashhad were analyzed by phylogenetic analysis performed on both *gag *and *env *subgenomic regions (approx. 300 bp), which have been repeatedly shown to be informative for an accurate subtype classification [14,15]. All Mashhad HIV-1 samples phylogenetically cluster with reference sequences of A subtype in *gag *as well as *env *subgenomic regions, showing no close phylogenetic relations with reference sequences of neither A sub-subtypes (A2, A3, A4) nor A-based circulating recombinant forms (Fig. 1A and 1B). Considering that most of the CRFs show a discordant phylogenetic classification in the *gag *and *env *subgenomic regions [20], the consistent clustering in the A subtype observed in the present study suggests the absence of intra-genomic recombination events which, however, need to be confirmed by near full-length sequence analyses. The Mashhad samples group in a single cluster indicating a strong phylogenetic correlation and a recent introduction of the HIV-1 infection in this community with a limited genetic evolution of the circulating virus. This observation is further confirmed by the overall low nucleotide divergence in *env *(10.8%, ± 2.39%) and *gag *(3.56%, ± 1.52%) sub-genomic regions of virus samples identified in the present cohort study. In particular, within the Mashhad cluster, a single variant pair (007 – 010) is observed in both *gag *and *env *phylogenetic trees. The genetic relationship between these two viral variants is further supported by the extremely limited nucleotide divergence (10.1% in *env *and 2.16% in *gag*), which strongly suggest a possible inter-personal direct transmission of the HIV-1 virus, although this is not confirmed by anamnestic data.

**Figure 2:**
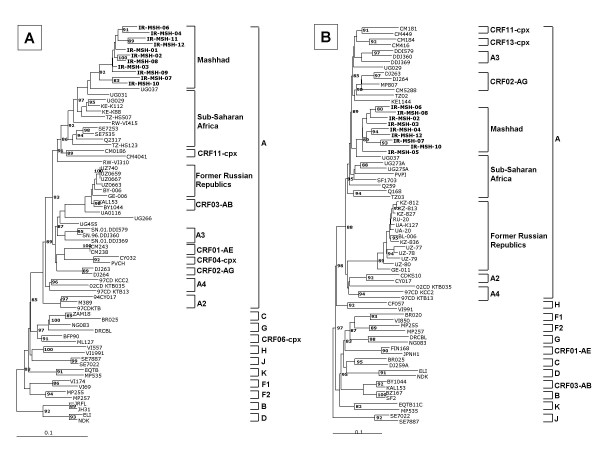
**Phylogenetic trees based on HIV-1 p24 gag (A) and C2-V3 env (B) regions including sequences from Eastern European and Middle-East Countries**. Nucleotide sequences of Mashhad isolates are compared with HIV-1 reference strains of Group M subtypes including a larger number of A-subtype reference sequences from Sub-Saharan African Countries along with sequences from Eastern European and Middle-East Countries. Trees have been constructed as in Fig. 1. The Mashhad sequences are indicated in bold characters. Sequence groups from different geographical regions are indicated.

In order to identify the possible geographic origin of the founder virus of HIV-1 epidemic in Mashhad, a phylogenetic analysis was performed including a larger number of A-subtype reference sequences from Sub-Saharan African Countries along with sequences from Eastern European and Middle-East Countries. Unexpectedly, the Mashhad cluster is closely related to the African Sub-Saharan viruses, with any phylogenetic correlation to the Eastern European and Middle-East variants. In particular, the Ugandan UG037 isolate is strongly correlated to the founder of the epidemic (Fig. 2A and 2B). This is further supported by the 100% homology in the amino acid composition of the V3 Loop tip between the consensus of Mashhad and sub-Saharan sequences (GPGQAFYAT), while the East European consensus sequence shows an A-to-T amino acid substitution (GPGQ**T**FYAT) [21].

These findings, although obtained in a small number of samples and, for each sample, on limited sub-genomic regions, suggest that the HIV-1 epidemic currently affecting the risk groups for HIV-1 infection in Iran (predominantly IDUs) is driven by viral variants of the A subtype. Furthermore, the strong phylogenetic correlation between the Mashhad and the sub-Saharan isolates (in particular Ugandan) supports the possibility that the founder virus has been introduced from African Countries more than from the neighboring Countries, which instead represent the main source of A-subtype variants in the whole European Continent. A similar phylogenetic link to African isolates of A-subtype variants identified in Tehran has been recently reported also by Sarrami-Forooshani *et al*., although a geographic origin from Former Soviet Union Countries has been proposed [22]. Considering the African origin of all A-subtype HIV-1 epidemics, this hypothesis could be supported by a HIV-1 speciation occurred at a different rate in the distinct regional epidemics, which, at this stage, is not supported by solid epidemiological data. Therefore, assuming a similar HIV-1 speciation rate in Iran and neighboring Countries, the phylogenetic pattern described in the present study, together with the results reported by Sarrami-Forooshani *et al*., would strongly suggest an independent and more recent direct introduction of African A subtype virus in Iran. This could possibly be correlated to the relevant role played by this Country, in particular Mashhad, in the Muslim religious culture.

These results on a sentinel cohort need to be confirmed by a nationwide molecular survey to verify the real distribution of A subtype in the country as well as in other risk groups (Homo- and heterosexuals groups). This molecular epidemiological information will be extremely relevant to guide the development and implementation of diagnostic as well as preventive/therapeutic approaches in Iran.
